# RS-1 enhances CRISPR/Cas9- and TALEN-mediated knock-in efficiency

**DOI:** 10.1038/ncomms10548

**Published:** 2016-01-28

**Authors:** Jun Song, Dongshan Yang, Jie Xu, Tianqing Zhu, Y. Eugene Chen, Jifeng Zhang

**Affiliations:** 1 Center for Advanced Models for Translational Sciences and Therapeutics, University of Michigan Medical Center, Ann Arbor, Michigan 48109, USA

## Abstract

Zinc-finger nuclease, transcription activator-like effector nuclease and CRISPR (clustered regularly interspaced short palindromic repeats)/Cas9 (CRISPR-associated protein 9) are becoming major tools for genome editing. Importantly, knock-in in several non-rodent species has been finally achieved thanks to these customizable nucleases; yet the rates remain to be further improved. We hypothesize that inhibiting non-homologous end joining (NHEJ) or enhancing homology-directed repair (HDR) will improve the nuclease-mediated knock-in efficiency. Here we show that the *in vitro* application of an HDR enhancer, RS-1, increases the knock-in efficiency by two- to five-fold at different loci, whereas NHEJ inhibitor SCR7 has minimal effects. We then apply RS-1 for animal production and have achieved multifold improvement on the knock-in rates as well. Our work presents tools to nuclease-mediated knock-in animal production, and sheds light on improving gene-targeting efficiencies on pluripotent stem cells.

## Abstract (web summary)

CRISPR/Cas9 and transcription activator-like effector nuclease (TALEN) are becoming major tools for genome editing. Here, Song *et al*. show that RS-1, a small-molecule enhancer for homology directed repair, increases the CRISPR/Cas9 and TALEN mediated knock-in efficiency both *in vitro* and *in vivo* with rabbit.

## Graphical abstract

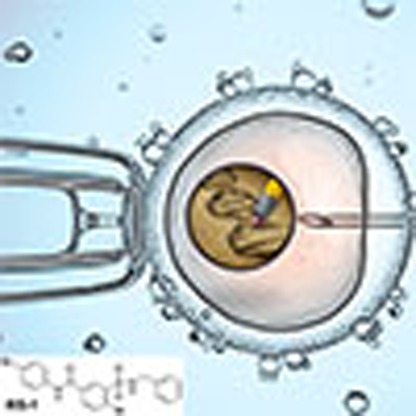

The advent of zinc-finger nuclease (ZFN), transcription activator-like effector nuclease (TALEN) and CRISPR (clustered regularly interspaced short palindromic repeats)/Cas9 (CRISPR-associated protein 9) technologies has changed the landscape of gene targeting. These customizable nucleases are efficient in generating double-strand breaks (DSB) in the genome that can lead to a functional knockout (KO) of the targeted gene or be used to knock-in a DNA sequence at a specific locus in the genome in a number of species^1^,^2^. In 2012, we produced *Apolipoprotein C3* (*ApoCIII*) KO rabbits using the ZFN approach^3^. In 2013, we successfully generated a number of KO rabbit lines using the Cas9 approach with high efficiencies^4^.

It is noted, however, that the efficiency of knock-in animal production, even with the help of these nucleases, remains low. Cui *et al* reported in 2011 that the success rates of ZFN mediated knock-in in mouse and rat embryos after pronuclear stage microinjection range from 0.3–2.2% (ref. 5). When TALEN was used in combination with oligodeoxynucleotides for microinjection to mouse embryos, the knock-in rate was 6.8% (1 knock-in founder out of 15 pups)^6^. Approximately, 15% pups contain knock-in alleles when Cas9 and donor DNAs were microinjected to mouse embryos by the Jaenisch group^7^,^8^. Our experience with the rabbit models confirmed these findings: the knock-in rates are below 1% when calculated as the ratio of total knock-in kits over total embryos transferred or 0–10% when calculated by ratio of total knock-in kits over total kits born. This low efficiency has become a rate-limiting factor for a broader application of nuclease-mediated gene modifications for transgenic animal production, as well as in pluripotent stem cells.

Non-homologous end joining (NHEJ) and homology-directed repair (HDR) are the two main mechanisms responsible for DNA repair after nucleases generate DSB at the target site^9^, where NHEJ would lead to KO characterized by unpredictable insertions or deletions (indels), whereas HDR results in knock-in events, when a donor vector is co-introduced. In the present work, we hypothesize that inhibiting NHEJ or enhancing HDR via small molecules will improve the nuclease-mediated homologous recombination (HR) efficiency. We have examined the effects of a potent NHEJ inhibitor, SCR7 (ref. 10), and an HDR enhancer, RS-1 (ref. 11), on improving the efficiency of Cas9- or TALEN-mediated knock-in in rabbits. We show that RS-1 enhances Cas9- and TALEN-mediated knock-in efficiency in rabbit embryos both *in vitro* and *in vivo*. Our work adds a useful tool to the transgenic animal research community and sheds light on regenerative medicine, as a substantial percentage of animal modelling and stem cell-based research and therapies will require targeted gene modifications.

## Results

In the context of this manuscript, ‘*in vitro*' embryos refer to the ones that were only cultured *in vitro* after microinjection and applicable treatments; ‘*in vivo*' embryos refer to the ones that were transferred to a pseudo pregnant recipient animal presumptively followed by implantation and fetal development.

### RS-1 increases Cas9-mediated knock-in efficiencies *in vitro*

We hypothesized that inhibiting NHEJ or enhancing HDR will increase the chance of knock-in events in the nuclease-mediated gene-targeting system.

We designed single guide RNA (sgRNA) targeting a *ROSA26*-like locus (*RLL*) in the rabbit genome (Fig. 2d). We next determined the effects of SCR7, an NHEJ inhibitor, and RS-1, an HDR enhancer on the knock-in success rates. Donor template DNAs were co-microinjected with Cas9 messenger RNA (mRNA) and sgRNA for *RLL* locus. After injection, the embryos were treated for 20 h in a serial concentration of SCR7 at 0 (control), 20, 40 and 80 μM, or in RS-1 at 0 (control), 7.5 and 15 μM.

**Figure 1:**
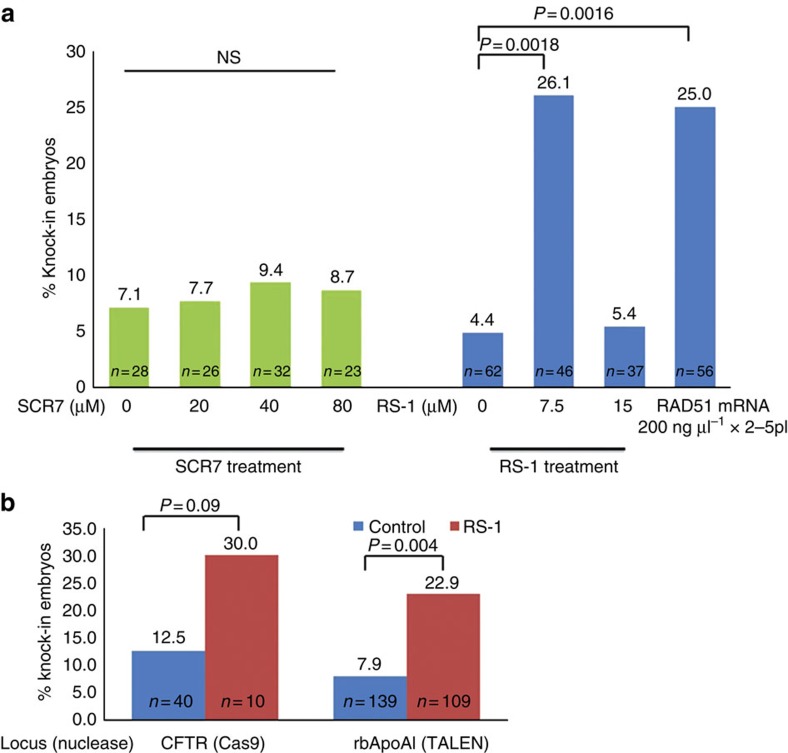
Effects of SCR7 and RS-1 on TALEN- or Cas9-mediated knock-in rates *in vitro*. (**a**) Efficiency of Cas9-mediated *RLL-EGFP* knock-in in rabbit embryos. Left panel: effects of SCR7. Right panel: effects of RS-1 or *RAD51* mRNA. (**b**) Effects of optimized RS-1 treatment (7.5 μM) on knock-in to *CFTR* and *ApoAI* loci in rabbit embryos versus control group (0 μM RS-1). Left panel: Cas9-mediated knock-in of CFTRdelF508 mutation to rabbit *CFTR*. Right panel: TALEN-mediated knock-in of *hApoAII* to *rbApoAI*. NS, not significantly different.

Treating embryos with SCR7 at these conditions had no effects on embryo development, as judged by blastocyst rates (62.1–71.8%, Supplementary Table 1). Furthermore, such treatments have no significant effects on the overall knock-in efficiency (7.1% in the control versus 7.7–9.4% in the treatment groups) (Fig. 1a).

Treating embryos with RS-1 at 15 μM appears to enhance the blastocyst development (82.2 versus 61.2% in the control, *P*<0.05, Fisher's least significant difference; Supplementary Table 1); but the knock-in efficiency was not improved (5.4 versus 4.8% in the control; Fig. 1a). Treating embryos with RS-1 at 7.5 μM, surprisingly, resulted in 26.1% (12 out of 46) knock-in rate, significantly higher (*P*<0.05, *χ*^2^-test) than those of control (4.4%) and at 15 μM (5.4%). The blastocyst development at this condition was 57.6%, similar to that of the control group.

**Figure 2:**
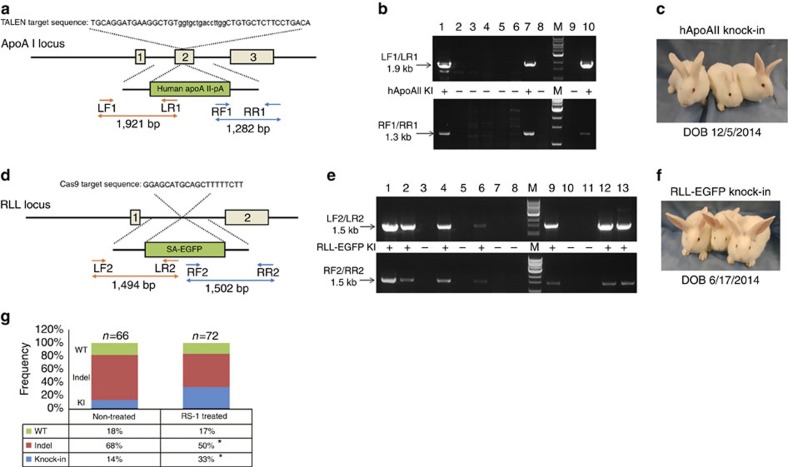
Effects of RS-1 on TALEN- or Cas9-mediated knock-in rates *in vivo*. (**a**) Gene-targeting strategy of TALEN-mediated knock-in of *hApoAII* to *rbApoAI* locus. (**b**) Confirmation of *hApoAII* knock-in rabbits. Lanes 1–10: samples from individual kits. Upper: PCR products using primer set LF1/LR1. Lower: PCR products using primer set RF1/RR1. M, molecule weight marker. Arrows indicate knock-in band. +, positive for knock-in. −, negative for knock-in. Kits #1, 7 and 10 are identified as knock-in founders. (**c**) Three *hApoAII* knock-in founder rabbits (DOB: 12/5/2014). (**d**) Gene-targeting strategy of Cas9-mediated knock-in of *EGFP* to *RLL* locus. (**e**) Confirmation of *RLL-EGFP* knock-in rabbits. Lanes 1–13: samples from individual kits. Upper: PCR products using primer set LF2/LR2. Lower: PCR products using primer set RF2/RR2. M, molecule weight marker. Arrows indicate knock-in band. +, positive for knock-in. −, negative for knock-in. Kits #1, 2, 4, 9, 12 and 13 are identified as knock-in founders. (**f**) Three *RLL-EGFP* knock-in founder rabbits (DOB: 6/17/2014). (**g**) Comparison of frequency of WT, indel and knock-in alleles between embryos parented by founders of the RS-1 treatment group and the non-treated group. *P<0.05, *χ*^2^-test.

To gain more confidence of using the RS-1 supplementation to improve nuclease-mediated knock-in efficiency in the embryos, we tested Cas9-mediated knock-in of CFTRdelF508, the most frequent mutation type identified in human cystic fibrosis patients, to the rabbit *CFTR* locus (Supplementary Fig. 2). We also ask the question whether the beneficial effects of RS-1 are restricted to the Cas9 system. We designed and validated TALEN pairs targeting *rbApoAI* locus for knocking in the *hApoAII*-coding sequence (Fig. 2a). Consistently, higher percentage of embryos carrying knock-in alleles were obtained in both the CFTRdelF508 (30 versus 12.5%, *P*=0.09, *χ*^2^-test) and *hApoAII* (22.9 versus 7.9%, *P*<0.05, *χ*^2^-test) cases when RS-1 supplementation was employed (Fig. 1b). These data indicate that RS-1 treatment work on different loci, and with different types of customizable nucleases.

### RS-1 improves Cas9-mediated knock-in efficiency *in vivo*

On the basis of *in vitro* results, we decided to use RS-1 at 7.5 μM for the *in vivo* experiments. The same sgRNA and donor DNA were used, for knocking in *EGFP* to the rabbit *RLL* (Fig. 2d,e).

In the control group, we transferred a total of 373 embryos to synchronized recipient rabbits and obtained 43 kits. Twenty-nine of these 43 kits (67%) carried indel alleles but with no knock-in alleles. Three are proven as knock-in founders after PCR and sequencing (Table 1). Of note, these three knock-in founders also carry indel alleles. The overall knock-in efficiency is 0.8% calculated by total knock-in founders/total embryos transferred or 7.0% calculated by total knock-in founders/total kits born.

In the RS-1 treatment group (Table 1), we transferred 146 embryos and obtained 38 kits, 18 of which carried indel but no knock-in alleles (47%). Similar to the *in vitro* results, the knock-in success rate was multifold higher than that in the control group (*P*<0.05, *χ*^2^-test). A total of 10 knock-in founders were produced, with the knock-in efficiency of 6.8% calculated by total knock-in founders/total embryos transferred or 26.3% calculated by total knock-in founders/total kits born. Again, all 10 knock-in founders carried indel mutations as well.

We considered the possibility of repetitive cleavage by the nucleases after knock-in. This would happen if the TALEN recognition sequences or the sgRNA target sequence remain intact after HR. Therefore, in the *CFTR* project, we introduced three silent mutations to the donor DNA (based on the seed region of guide RNA sequence) to prevent this from happening (Supplementary Fig. 2a). In the *in vivo* cases (for example, *RLL-EGFP* and *hApoAII*), the TALEN recognition sequences and the sgRNA target sequence were replaced by a untargetable knock-in sequence; therefore, there is low possibility of NHEJ errors within the knock-in allele. Nevertheless, we performed molecular cloning, PCR and sequencing on all founder animals (*n*=13 for *RLL-EGFP* and *n*=4 for *hApoAII*). In particular, we looked at the left and right adjacent regions of the knock-in sequences (Supplementary Fig. 4a), as these ‘linking' regions are the most likely places where HR errors may take place. All of the founder animals have correct sequences in the left adjacent regions and right adjacent regions in the knock-in alleles (representative sequencing results shown in Supplementary Fig. 4b,c). We did not identify any errors in the knock-in sequences either, as expected. These results indicate that there are no additional NHEJ-mediated errors introduced to the knock-in alleles, with or without RS-1 treatment, in these founder rabbits.

### RS-1 improves TALEN-mediated knock-in efficiency *in vivo*

We also tested RS-1 on the TALEN system *in vivo*. The same TALEN pairs and donor DNAs used in the *in vitro* work were used to product *hApoAII* knock-in animals (Fig. 2a,b).

Without RS-1 treatment, only one founder animal was produced out of 227 embryos transferred (Table 1). The knock-in efficiency was 0.4% calculated by total knock-in founders/total embryos transferred or 6.3% calculated by total knock-in founders/total kits born.

After RS-1 treatment, we transferred a total of 145 embryos and obtained 17 kits. Seven out of 17 kits carried indel but no knock-in alleles (41%). Three rabbits were confirmed as knock-in founders (Table 1), resulting in the knock-in efficiency of 2.1% calculated by total knock-in founders/total embryos transferred or 17.6% calculated by total knock-in founders/total kits born, multifold higher than the control group. Again, all knock-in founders, regardless of being treated with RS-1 or not, carried indel mutations as well.

These results indicate that RS-1 is effective on improving knock-in rates in both the TALEN- and the Cas9-mediated genome-editing systems.

### Beneficial effects of RAD51 mRNA

RAD51 recombinase is a key player for HR and the repair of DNA DSBs^12^. Since it has been reported that RS-1 stimulates RAD51 (ref. 11), we reasoned that including RAD51 mRNA in the microinjection mixture would yield similar improvements on the knock-in efficiency as achieved with RS-1.

We microinjected 79 embryos with *RAD51* mRNA, in addition to sgRNA, Cas9 mRNA and donor DNAs (Supplementary Table 1). Fifty-six of these embryos (71%) developed to blastocyst stage, higher than those in the control group. All 56 blastocysts were PCR and sequenced, out of which 14 are knock-in positive (25%), significantly higher (*P*<0.05, *χ*^2^-test) than that in the control group (4.4%) (Fig. 1a).

These results suggest that co-microinjection of *RAD51* mRNA may be used in substitution of RS-1 treatment to simplify the procedure. It also suggests that RS-1 likely functions through stimulating RAD51 to enhance the nuclease-mediated knock-in efficiency.

### Germline transmission of the *RLL-EGFP* founder rabbits

No abnormalities are found in both the *RLL-EGFP* and *hApoAII* founder animals derived from both the RS-1 treatment and control groups (Fig. 2c,f). At the time of submitting this manuscript, five *RLL-EGFP* founders, three from the RS-1 treatment group and two from the non-treatment group, have reached sexual maturity. We bred these *RLL-EGFP* founder animals with WT counterparts.

Sixty-six embryos parented by the founders of the non-treatment group were collected (Table 2; Fig. 2g). Nine embryos carried the knock-in allele (14%); however, all came from the same founder (#10747). The knock-in alleles in the other founder (#10588) appears not germline transmitting.

Seventy-two embryos parented by the founders of the RS-1 treatment group were collected (Table 2; Fig. 2g). Twenty-four embryos carried the knock-in allele (33%). Notably, all three founders (#10245, 10247 and 10244) in this group germline transmitted the knock-in allele.

We also allowed one pregnancy fathered by founder animal #10245 to full term, resulted in eight kits, four of which (50%) carry the *RLL-EGFP* knock-in genotyping, and as expected, all expressed EGFP consecutively.

In sum, RS-1 treatment does not appear to have any toxic effects on the overall animal health and reproduction. All three animals generated from the RS-1 treatment group are germline transmitting (100%); whereas only one of the two from the non-treatment group is germline transmitting (50%), indicating that RS-1 does not adversely affect, if not improves, the germline transmitting capacity of the knock-in founder animals.

### RS-1 treatment enhances HR frequencies

We have shown that at the whole-organism level, RS-1 treatment enhanced the chance of obtaining founder animals carrying the knock-in allele (Table 1). We further ask the question whether the frequency of knock-in alleles, particularly in the germline cells, is altered after RS-1 treatment. Like we described above, to ultimately determine the frequencies of different types of alleles (that is, WT, indel and knock-in), we used WT animals to breed with five *RLL* knock-in founders, two derived without RS-1 treatment and the other three with RS-1 treatment.

Embryos were produced from founders of the RS-1-treated group (n=72) and the non-treated group (*n*=66), sequenced and categorized as WT, indel or knock-in based on the allele sequence (Fig. 2g). Similar percentage of WT alleles (17 versus 18%) are found between these two groups. Notably, consistent with the findings at the whole-organism level, the knock-in frequency at the allele level was significantly higher (*P*<0.05, *χ*^2^-test) in the RS-1-treated group (33%) than in the non-treated group (14%). However, it should be noted that the results are obtained from a relatively small number of animals (*n*=5); additional experiments are necessary to determine whether the knock-in events take place at the expense of KO.

These data show that in addition to enhancing the chance of obtaining the knock-in founder animals, RS-1 treatment further increased the percentage of knock-in alleles in the derivative embryos/animals. These two factors multiply to result in the significantly improved efficiency of knock-in animal production.

## Discussion

ZFN, TALEN and CRISPR/Cas9 are efficient in generating DSBs in the genome that can lead to a functional KO of the targeted gene or used to integrate a DNA sequence at a specific locus in the genome in a number of species^1^,^2^. These nuclease-based genome-editing systems are becoming the mainstream tools for gene targeting in animal embryos and cells, especial non-murine species including humans, thanks to its high efficiency and ease of use. Two major mechanisms, namely, NHEJ and HDR, function to repair DSBs. As its name suggests, in NHEJ, the break ends are directly ligated without the need for a homologous template, thus leading to generally unpredictable indels at the targeting locus. HDR may take place, in addition to NHEJ, when homologous donor templates are present, leading to correct repair or knock-in events.

After the NHEJ repair, if the resulting indels cause frameshift mutations, functional KO may be observed in the derivative animals and cells. The Cas9-mediated KO rates are very high in a wide spectrum of species, from zebrafish^13^ and drosophila^14^ to mouse^7^, rat^15^, rabbits^4^ and humans cells^16^. We previously reported the production of KO rabbits using the ZFN and Cas9 system^3^,^4^. The KO rates using the Cas9 system range from 10 to 100% *in vitro* and 32.1 to 83.3% *in vivo*^4^. However, the frequency of HDR appears to be much lower than that of NHEJ. Without any intervention, the HDR/NHEJ ratio calculated by the number of indel events over that of knock-in events is below 10% in our rabbit system, consistent with reports in other species. For example, Gonzalez *et al* reported 2–3% HDR rates versus 13–49% indel rates in human embryonic stem and induced pluripotent stem cells in 2014 (ref. 17). Likewise, in one mouse study, the NHEJ-mediated gene editing is 28–50%, whereas the HDR-mediated knock-in is below 10% (ref. 7). Collectively, the HDR events take place at 1/3 or even lower frequencies than the NHEJ events.

Such low knock-in rate has become a bottleneck problem for the broad application of the Cas9 and other customizable nuclease systems in biomedical research, because for reliable disease modelling and gene correction it is often necessary that a specific change be introduced to the sequence. Even for gene addition therapy, it is desirable that such addition is location and copy-number controlled, which has been demonstrated by knock-in to the *ROSA26* or similar safe harbour locus.

Jayathilaka *et al*. reported in 2008 that RS-1 (3-((benzylamino) sulfonyl)-4-bromo-*N*-(4-bromophenyl) benzamide) stimulates the human HR protein RAD51, a key player in the HR complex^11^, suggesting RS-1 as a good small-molecule candidate to improve HR efficiency in embryos and cells mediated by nucleases. Indeed, our results show for the first time that RS-1 can increase both TALEN- and Cas9-mediated knock-in efficiencies in the rabbit system. The *in vivo* knock-in efficiencies were improved approximately two- to fourfold in two genes that we targeted. The likely mechanism of such improvement is through RS-1 stimulating *RAD51*, because co-microinjection of *hRAD51* mRNA replicated the RS-1 treatment outcome. Consistent with this, overexpression of RAD51 in different organisms and cell types generally increased HR^12^. We speculate that modulating RAD51-interacting factors such as PALB2 (partner and localizer of BRCA2)^18^, Nap1 (nucleosome assembly protein 1)^19^, p400 ATPase^20^, EVL (Ena/Vasp-like)^21^ and so on may also lead to enhanced HR frequencies in nuclease-mediated gene targeting. One should also keep in mind that in one study Paffett *el al*. showed that the overall rate of all types of HR events were reduced when RAD51 was overexpressed at high levels of at least a 10-fold increase over normal levels in *Saccharomyces cerevisia*^22^, implicating that species-specific response, as well as dosage and temporal effects should be considered in any efforts to exploiting this pathway to achieve high HR rates. The pronuclear microinjection of *RAD51* mRNA method may be advantageous because of the precise location of delivery, the active recombinant events at this embryonic stage and the short action window as compared with prolonged expression achieved by viral transduction.

It is noted that the toxic effects of RS-1 appears minimal in our rabbit system: (i) the blastocyst rates were similar or higher in the supplementation groups than in the blank control group; (ii) the founder animals produced from the RS-1 treatment groups all appear healthy and indistinguishable from the founders produced from the non-treatment group; and (iii) the F1 offspring generated from one founder animal from the RS-1 group are all healthy.

Surprisingly, however, NHEJ inhibitor SCR7 showed no significant effects on improving the overall HDR events in our rabbit system. Nor has it lowered the NHEJ frequencies as measured by indel rates (Supplementary Fig. 1). This may suggest that alternative NHEJ mechanisms that are not SCR7 sensitive exist in rabbits. Such ineffectiveness is different from several recent reports in which supplementation of SCR7 increased the HDR frequencies by as much as 10-fold in mouse embryos and mammalian cells^23^,^24^,^25^. Additional work is needed to confirm the effects of SCR7 and/or other NHEJ inhibitors in our system because only a small number of embryos were used to target a single gene in the present work. The discrepancy between our work and these reports suggest that different species may respond differently to these treatments, which consequently necessitate future work to examine effects of RS-1 in mouse and human systems. Equally important, the toxicity effects of both NHEJ inhibitors and HDR enhancers must be fully addressed. Severe cell death was observed in our work testing SCR7 on human induced pluripotent stem cells.

It also came to our attention that we used large size donor DNAs (3–5 kb) in all knock-in projects in the present work. It remains to be tested whether RS-1 has similar effects when double stranded oligodeoxynucleotide (dsODN) or single stranded oligodeoxynucleotide (ssODN) (<200 bp) is used as a donor template.

Taken together, our work provides a potential solution to the rate-limiting problem, that is, low knock-in efficiency associated with nuclease-based gene targeting. It is proven effective in both the Cas9 and TALEN systems, elevating the knock-in rates by multifold. With improved success rates, founder knock-in rabbits can be produced in as few as one embryo transfer experiment, markedly saving man hours and reducing animal uses. The present work and several recent publications demonstrate that inhibiting NHEJ or enhancing HDR is a viable approach to improve HR efficiencies in animal embryos, pluripotent stem cells and other mammalian systems^24^,^25^,^26^. These strategies, in combination with other approaches such as synchronization of cell cycles^27^, will further enhance the applicability of customizable nucleases, as a substantial percentage of stem cell-based therapies will require targeted gene modifications. Moreover, enhanced knock-in efficiency may enable genetic modifications on both alleles, and even multiplex gene corrections in embryos and pluripotent stem cells.

## Methods

### CRISPR/Cas and TALEN plasmids construction and RNA synthesis

For CRISPR/Cas9-mediated knock-in experiments, the Cas9 expression plasmid JDS246 and sgRNA expression plasmid DR274 were obtained from Addgene. sgRNA was designed using Zifit software (http://zifit.partners.org/ZiFiT/), synthesized and cloned into the plasmid DR274. The targeted sequences for RLL and CFTR are shown in Fig. 2d and Supplementary Fig 2, respectively.

For TALEN-mediated knock-in experiments, TALEs were designed using online software (https://tale-nt.cac.cornell.edu/node/add/talen). The Golden Gate TALEN and TAL Effector Kit 2.0 used for the generation of TALENs was a gift from Daniel Voytas and Adam Bogdanove (Addgene kit # 1000000024)^28^. TALEN plasmids were constructed according to the kit instruction. Briefly, single DNA-binding repeat modules were assembled into the intermediary arrays of 1–10 repeats; then joining of the intermediary arrays into the final destination vector pTALEN-GG-L or pTALEN-GG-R, containing the modified FokI nuclease domain. The targeted sequence for rbApoAI is shown in Fig. 2a.

For *RAD51* mRNA co-injection experiments, the full-length Human *Rad51* cDNA (accession#: NM_002875) was amplified by PCR and cloned into pcDNA3.1vector (Invitrogen).

TALEN, Cas9 and *RAD51* mRNAs were transcribed *in vitro*, caped and polyadenylated using the T7 mScript Standard mRNA Production System (C-MSC100625, CELLSCRIPT, Madison, WI). sgRNA was *in vitro* transcribed using T7-Scribe Standard RNA IVT Kit (C-AS3107, CELLSCRIPT). TALEN mRNAs, Cas9 mRNA, *RAD51* mRNA and sgRNA were diluted in RNase-free TE buffer (1 mM Tris-Cl pH 8.0, 0.1 mM EDTA), stored in −80 °C in 10 μl aliquots, and were thawed and kept on ice before microinjection.

### Construction of HR donor vectors

HR donor vectors were constructed by standard molecular-cloning methods. For RLL locus, the donor vector consists of the 0.5-kb 5′-homology arm and the 0.7-kb 3′-homology arm, flanking an adenoviral splice acceptor sequence, followed by the 2-kb *EGFP* expression cassette (Fig. 2b). For *CFTR* locus, the donor vector consists of the 2.4-kb 5′ homology arm and the 1.6-kb 3′ homology arm, flanking delF508 mutation (Supplementary Fig. 2a). For *rbApoAI* locus, the donor vector consists of the 1-kb 3′ homology arm, *hApoAII*-coding sequence and 1 kb 5′-homology arm (Fig. 2a).

### Microinjection and embryo transfer

All animal maintenance, care and use procedures were reviewed and approved by the University Committee on the Use and Care of Animals of the University of Michigan. Sexually matured (6–18 months) New Zealand White female rabbits were superovulated by subcutaneous injection of follicle-stimulating hormone (Folltropin-V, Bioniche Life Sciences, Canada) twice per day with a dosage of 3 mg for the first two injections, 5 mg for the next two injections and 6 mg for the last two injections. Seventy-two hours after the first follicle-stimulating hormone injection, a single intravenously injection of 200 IU human chorionic gonadotropin (Chorulon, Intervet, Holland) was administered to induce ovulation. The superovulated females were mated with male rabbit immediately after human chorionic gonadotropin injection. Sexually matured recipient female rabbits will be synchronized by stimulate mechanically in the vagina and injection of 0.3 ml gonadotropin-releasing hormone agonist (Receptal, Merck animal health) intramuscularly. Eighteen hours post insemination, the superovulated rabbits were killed. The oviduct ampullae were recovered, flushed with 10 ml of HEPES-buffered manipulation medium containing 25 mM HEPES-buffered TCM 199 (#12350039, Life Technologies, Grand Island, NY) supplemented with 10% fetal bovine serum (#12003C, Sigma, St. Louis, MO) and the recovered oocytes were observed under a microscope for the occurrence of fertilization, and then kept in the HEPES-buffered manipulation medium at 38.5 °C in air.

Microinjection was performed on pronuclear stage embryos 19–21 h post insemination using a micromanipulator under the inverted microscope equipped with a differential interference contrast device. Rabbit embryo was held with a holding glass pipette (120–150-μm diameter) in HEPES-buffered manipulation medium. A mixture containing 100 ng μl^−1^ donor DNA (hApoAII) and 50 ng μl^−1^ each of the TALEN mRNAs, or a mixture containing 100 ng μl^−1^ donor DNA (*RLL-EGFP* or CFTRdelF508), 100 ng μl^−1^ Cas9 mRNA and 6 ng μl^−1^ sgRNA, was used for cytoplasm microinjection. For *RAD51* co-injection, *RAD51* mRNA was added into the mixture in a final concentration of 200 ng μl^−1^. Approximately, 2–5 pl mixture was injected to each embryo. Injected embryos were washed three times in embryo culture medium, which consisted of Earle's Balanced Salt Solution (E2888, Sigma) supplemented with non-essential amino acids (M7145, Sigma), essential amino acids (B-6766, Sigma), 1 mM L-glutamine (25030-081, Life Technologies), 0.4 mM sodium pyruvate (11360-070, Life Technologies) and 10% fetal bovine serum. The injected embryos were cultured overnight (20 h) with or without SCR7 or RS-1 treatment before surgically transferred into the oviduct of a synchronized recipient doe. Embryos (20–30) were transferred to one recipient doe. For *in vitro* validation, instead of transferring to recipient doe, the injected and treated embryos were washed and cultured in medium without RS-1 or SCR7 *in vitro* for additional 3–4 days until they reach blastocyst stage.

### Confirmation of gene-targeting events

For *in vitro* validation, blastocyst stage embryos were lysed individually and genomic DNA extracted. To get better PCR reaction, the whole genome was replicated using a REPLI-g Mini Kit (Qiagen, Germantown, MD) following the manufacturer's protocol with slight modification. Briefly, for collected denatured DNA, 3.5 μl buffer D2 was added to each embryo, mixed by vortexing and centrifuged briefly. The samples were incubated on ice for 10 min. After that, 3.5 μl stop solution was added, mixed by vortexing and centrifuged briefly. For replication, 2 μl of the denatured DNAs were added to 8 μl master mix and incubate at 30 °C for 10–16 h. Then, REPLI-g Mini DNA polymerase was inactivated by heating at 65 °C for 3 min.

To determine genotypes of founder animals, ear skin tissues were biopsied and genomic DNA was extracted. Genomic DNAs were next used for PCR using corresponding primers (Supplementary Table 2). PCR products were analysed with agarose gel electrophoresis for knock-in detection (for *RLL-EGFP* and *hApoAII*) or purified and sequenced for detection of indel mutations and knock-in of the CFTRdelF508. On the chromatographic curves, peaks on peaks approximate the targeting site indicate an indel event. For hApoAII, a knock-in event will show a 1.9-kb band from the LF1/LR1 primer pair and a 1.3-kb band from the RF1/RR1 primer pair (Fig. 2a,b). For *RLL-EGFP*, a knock-in event will show a 1.5-kb band from the LF2/LR2 primer pair and a 1.5-kb band from the RF2/RR2 primer pair (Fig. 2d,e). For CFTRdelF508, a knock-in event cannot be resolved by agarose gel banding, due to trivial size difference. The PCR products were purified and sequenced; reading of delF508 indicates knock-in (Supplementary Fig. 2b). Mutation Surveyor (Softgenetics, State College, PA) was used to analyse sequencing results.

### Germline transmission and allele types

*RLL-EGFP* founder rabbits were bred with WT counterparts. Embryos were collected as described in the section Microinjection and embryo transfer. All embryos were genotyped as described in the section Confirmation of gene-targeting events, and subsequently categorized to ‘WT', ‘indel' and ‘knock-in', based on the mutation types on the alleles inherited from the founder animal. Identification of knock-in embryos indicates the germline transmitting capacity of the corresponding founder animal.

### Statistical analysis

*χ*^2^-test in Prism (Graphpad Software, Inc., La Jolla, CA) was used to calculate *P* values when comparing the knock-in rates, indel rates or the blastocysts rates in embryos or animals between the treatment group (for example, SCR7 or RS-1 treated) and the control (0 μM). For example, the embryos or animals were categorized as ‘knock-in' or ‘non-knock-in' (including indels and WT) for comparison of knock-in efficiencies. Embryo development rates were transformed using arcsin transformation, analysed by analysis of variance and means compared by Fisher's least significant difference using Graphpad Prism. Significant differences were defined as *P*<0.05 (*).

## Additional information

**How to cite this article:** Song, J. *et al*. RS-1 enhances CRISPR/Cas9- and TALEN-mediated knock-in efficiency. *Nat. Commun.* 7:10548 doi: 10.1038/ncomms10548 (2016).

## Supplementary Material

Supplementary Information Supplementary Figures 1-4 and Supplementary Tables 1-2

## Figures and Tables

**Table 1:** Summary of knock-in animal production.

**Nuclease**	**RS-1**	**Target**	**No. of embryos transferred**	**No. of kits**	**Kits with indels but no KI**	**Kits with KI**	**KI per embryo (%)**	**KI per kit (%)**
TALEN	−	*rbApoAI*	227	16	11	1	0.4	6.3
TALEN	+	*rbApoAI*	145	17	7	3	2.1*	17.6
Cas9	−	*RLL*	373	43	29	3	0.8	7.0
Cas9	+	*RLL*	146	38	18	10	6.8**	26.3**

Cas9, CRISPR-associated protein 9; indels, insertion and deletions; KI, knock-in; TALEN, transcription activator-like effector nuclease.

Comparisons were made between RS-1 supplemented group (+) and non-treatment group (−) of the same locus. *P<0.05, **P<0.01.

**Table 2:** Summary of germline transmission and allele distribution in embryos produced by *RLL-EGFP* founders.

**Founder ID#**	**RS-1 trt**	**No. of F1 embryos**	**Alleles from founder**			**KI germline transmission**
			**Indel allele**	**KI allele**	**WT allele**	
10747	No	29	16	9	4	Yes
10588	No	37	29	0	8	No
10245	Yes	20	1	13	6	Yes
10247	Yes	11	6	2	3	Yes
10244	Yes	41	29	9	3	Yes

Indel, insertion and deletion; KI, knock-in; WT, wild type.
