# Supplementary material for: RS-1 enhances CRISPR/Cas9- and TALEN-mediated knock-in efficiency
Source: Nat Commun. 2016 Jan 28;7:10548. doi: 10.1038/ncomms10548 (PMC4738357; doi:10.1038/ncomms10548)
Supplement: Supplementary Information — Supplementary Figures 1-4 and Supplementary Tables 1-2 [file ncomms10548-s1.pdf]

Supplementary Information

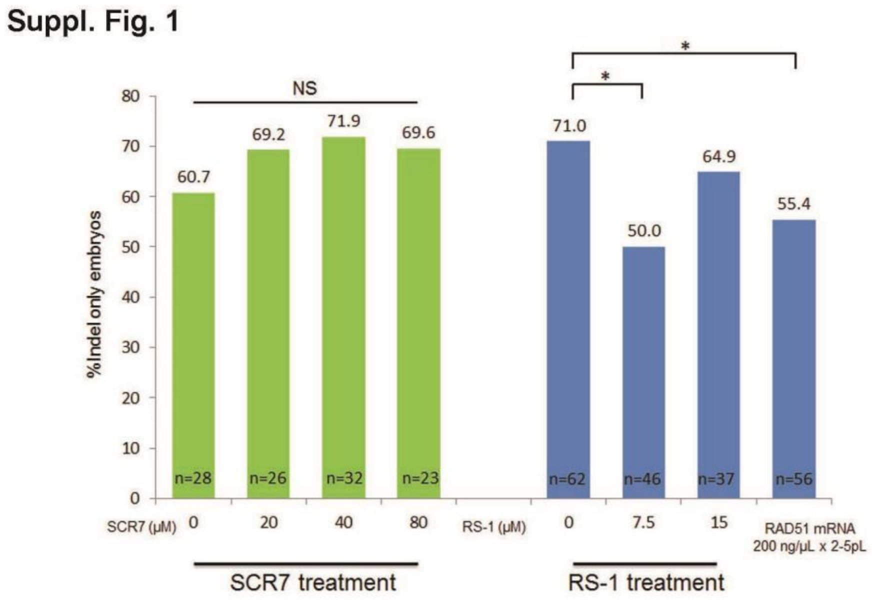

Suppl. Fig. 1. **Effects of SCR7 and RS-1 on the frequency of indel-only embryos.** NS: not significant. *P<0.05.

Suppl. Fig. 2

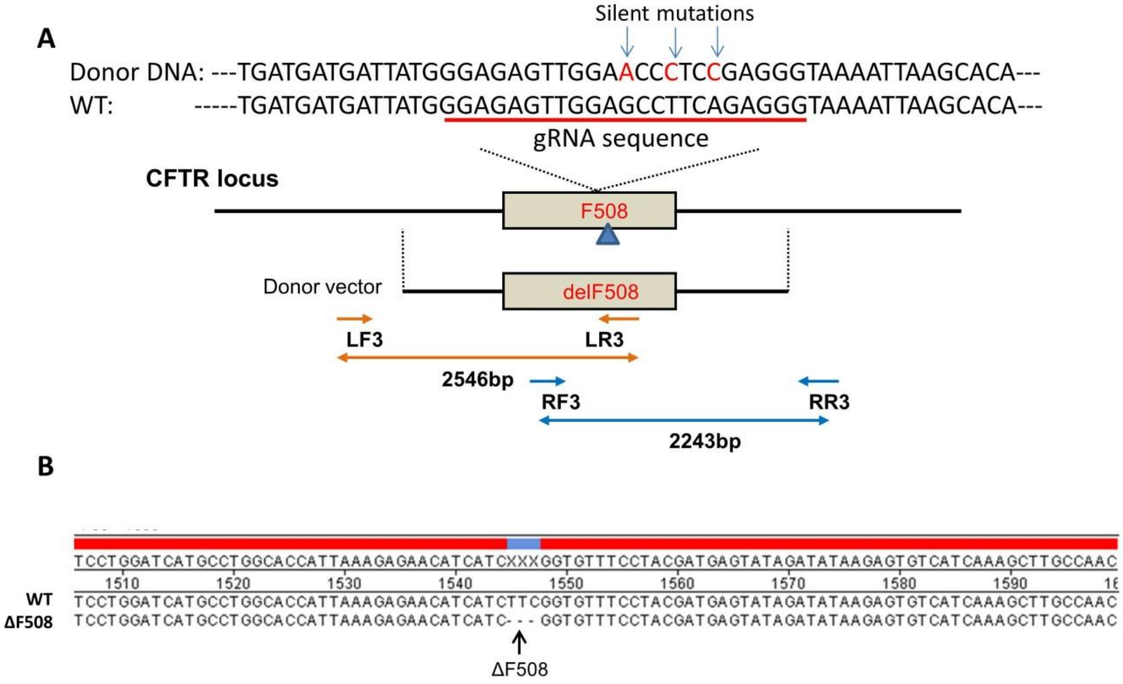

Suppl. Fig. 2. **Knock-in of CFTRdelF508 mutation to rabbit genome.** (A) Gene targeting strategy of Cas9 mediated knock-in of CFTRdelF508 to CFTR locus in rabbit genome. Silent mutations (arrows) were made in the donor template to prevent repetitive cleavage by Cas9 after knock-in. LF3, LR3, Rf3, RR3 are primers used for PCR. Sequences of these primers are available in Suppl. Table 1. (B) A representative sequencing result showing successful knock-in of the CFTRdelF508 mutation.

Suppl. Fig. 3

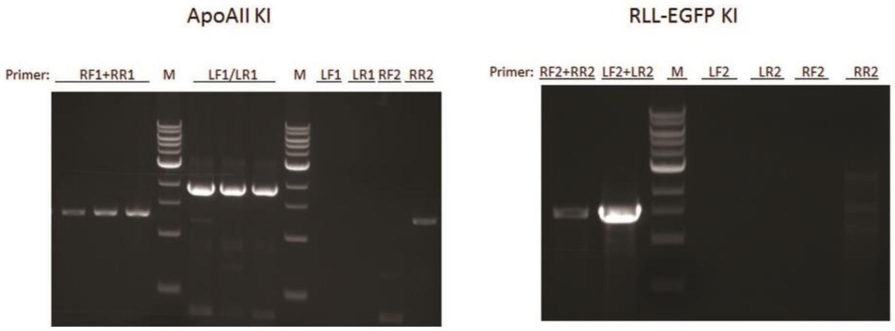

Suppl. Fig. 3. Validation of the PCR assays using single primers. Left: PCR results by using primers pairs (RF1+RR1, or LF1+LR1) or single primers (LF1, LR1, RF2 or RR2) on an ApoAII knock-in founder. KI: knock-in. M: molecular weight marker. Knock-in specific bands were detected in both primer pairs (in triplicates), but not in single primer lanes. A non-specific band was present in the RR2 lane. Right: PCR results by using primers pairs (RF1+RR1, or LF1+LR1) or single primers (LF1, LR1, RF2 or RR2) on an RLL-EGFP knock-in founder. KI: knock-in. M: molecular weight marker. Knock-in specific bands were detected in both primer pairs, but not in single primer lanes.

Suppl. Fig. 4

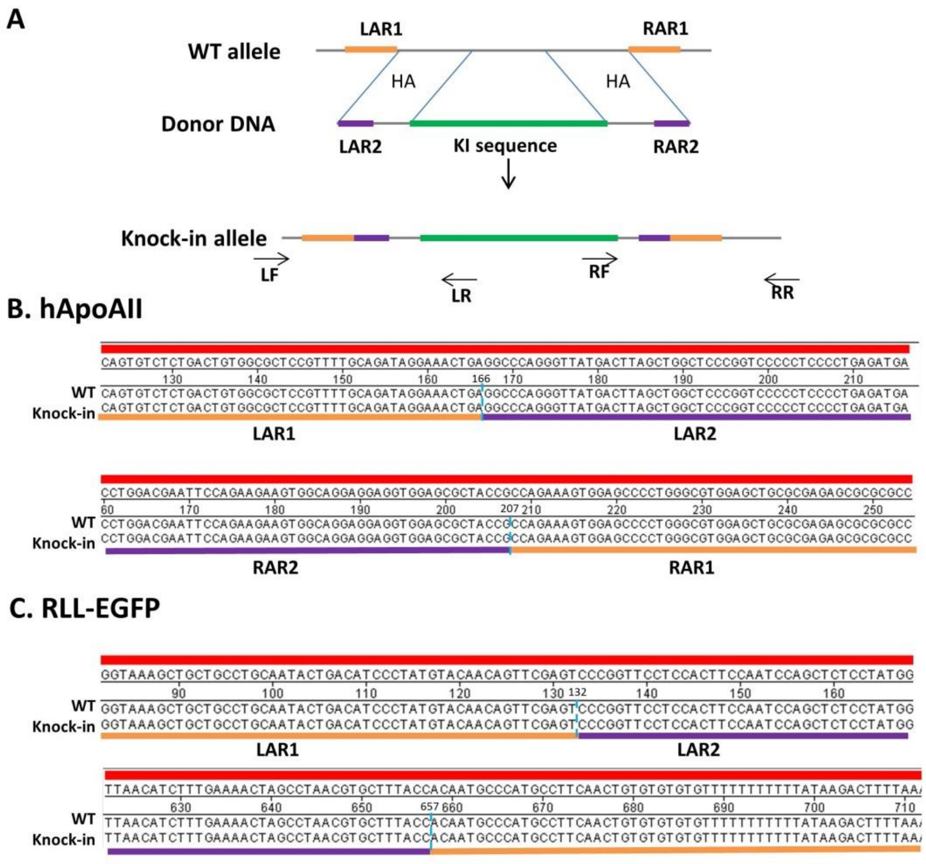

Suppl. Fig. 4. Correct incorporation of knock-in sequences in RLL-EGFP and hApoAII founder rabbits. (A) Illustration of the general strategy of molecular cloning followed by PCR and sequencing to confirm correct incorporation of the knock-in sequences in RLL-EGFP (n=13) and hApoAII (n=4) founder rabbits. Orange sequences (LAR1 and RAR1) refer to adjacent regions to the HR linking loci on the WT allele. Purple sequences (LAR2 and RAR2) refer to adjacent regions to the HR linking loci on the donor template. Green sequence refers to the knock-in sequence. PCR were conducted using LF/LR and RF/RR primer pairs, followed by sequencing to obtain LAR and RAR sequences. Correct incorporation of the knock-in allele will be indicated by the identical sequences of the LAR1-LAR2 and RAR2-RAR1 regions in the knock-in allele

comparing to the WT allele. LAR: left adjacent region. RAR: right adjacent region. KI: knock-in. LF: left forward primer. LR: left reverse primer. RF: right forward primer. RR: right reverse primer. (B) Representative sequencing results of LARs and RARs in a founder hApoAII rabbit. The linking base is at location #166 and #207 for LARs and RARs, respectively. The knock-in allele contains identical sequence to the WT allele, indicating correct incorporation of the knock-in sequence. (C) Representative sequencing results of LARs and RARs in a founder RLL-EGFP rabbit. The linking base is at location #132 and #657 for LAR and RAR, respectively. The knock-in allele contains identical sequence to the WT allele, indicating correct incorporation of the knock-in sequence.

Supplementary Table 1. Effects of SCR7 and RS-1 on embryo development.

Trt	Dosage	Embryo injected	Blastocysts (%)
**SCR-7**	0 uM	43	28 (65.1)
	20 uM	39	28 (71.8)
	40 uM	58	36 (62.1)
	80 uM	37	24 (64.9)
**RS-1**			
	0 uM	103	63 (61.2%)
	7.5 uM	85	49 (57.6%)
	15 uM	45	37 (82.2%)*
**RAD51 mRNA**	200 ng/μL x 2-5pL	79	56 (70.9%)

Supplementary Table 2. Primers used for confirmation of knock-in events.

Primers used for hApoAII knock-in experiment
RF1: 5’-TCTCTGACTGTGGCGCTCCGTTTTG-3’
RR1: 5’-ACCAGTTCCGTTCCAGCCTTCTTGAT-3’
LF1: 5’-CGGCCGCGGTCATAGCTGTTTCCTG-3’
LR1: 5’-CGCCGCCGCCCTCCTTGATG-3’
Primers used for RLL-EGFP knock-in experiment
RF2: 5’-AGGTGAGAAACAGGCAGAAATAGT-3’
RL2: 5’-TGTCCAAACTCATCAATGTATCTTA-3’
LF2: 5’-AGCCCTAAATTCAAGCCCTGTG-3’
LR2: 5’-GGAAACCCTGGACTACTGCG-3’
Primers used for CFTRdelF508 knock-in experiment
LF3: 5’-GCTTTATGGTTCCCTTACGGTTTA-3’
LR3: 5’-ATTATGGGAGAGTTGGAGCCTTCA-3’
RF3: 5’-ATACACCAACCTAGCCCATCATT-3’
RR3: 5’-GACTTAACCTGCTTCACCACAA-3’
